# Longitudinal data on (political) news consumption and political attitudes in a German sample collected during the election year 2021

**DOI:** 10.1016/j.dib.2022.108326

**Published:** 2022-05-29

**Authors:** Cornelia Sindermann, Christopher Kannen, Christian Montag

**Affiliations:** a Institute of Psychology and Education, Ulm University, 89081 Ulm, Germany; b Department of Psychology, University of Toledo, Toledo 43606, OH, USA

**Keywords:** News Consumption, Filter Bubble, Echo Chamber, High Degree of Homogeneity in News Consumption, Voting Intentions, Political Attitudes

## Abstract

The present data set contains self-report data of German individuals participating in a longitudinal data assessment via online surveys conducted in the year preceeding the general elections in Germany.

Data of N = 122 individuals are included in the data set. Those individuals participated in an initial, extensive survey between November 2020 and February 2021 (T1) as well as in a final survey after the general German elections, thus, between the end of September 2021 and October 2021 (T3). Of those individuals, n = 93 additionally participated in an intermediate survey in between the previously mentioned ones between the end of May and the end of June 2021 (T2).

Next to the assessment of sociodemographic variables, information on (political) news consumption, such as the frequency of being confronted with counter-attitudinal news, and on political attitudes, for example via current voting intentions for one of the major German parties, were assessed in the initial survey (T1).

In the intermediate survey (T2), participants provided information on recent political news consumption habits including the frequency of being confronted with counter-attitudinal news, current voting intentions for one of the major German parties, as well as on extraordinary events that happened recently and impacted their voting intentions.

In the final survey (T3), sociodemographic variables and actual voting decisions in the general German elections in 2021 were assessed. Moreover, variables on recent political news consumption habits, including the frequency of being confronted with counter-attitudinal news, and extraordinary events that happened recently and impacted voting decisions were assessed. Finally, a detailed self-report questionnaire retrospectively assessing political news consumption for the time between participation in the initial survey (T1) and the final survey (T3) was completed by participants. Not only did this questionnaire assess which online and offline news channels (e.g., TV, print, news websites) participants used. Besides, the questionnaire included items on how many outlets per channel were used and the frequency of being confronted with counter-attitudinal news within each channel.

This data set is provided alongside the present article to be used for further investigations of the stability of voting intentions, thus, political attitudes. Moreover, a content analysis of the open responses on which extraordinary events happened and impacted voting intentions/decisions can provide further knowledge on factors influencing voting intentions and their variability versus stability.

## Specifications Table

**Table:** 

Subject	Political Science
Specific subject area	Longitudinally assessed self-report data on (political) news consumption and voting intentions/decisions in a German sample in 2021
Type of data	Table (Excel)
How the data were acquired	Data were collected in three online surveys programmed on the SurveyCoder platform (https://www.surveycoder.com/; https://ckannen.com/) between i) November 2020 and February 2021 (T1), ii) May and June 2021 (T2), ii) September and October 2021 (T3). Participants could complete the surveys from any electronic device connected to the internet. Participants were invited via email and pseudonymous individual codes were used to match data across measurement times/surveys. All participants provided informed electronic consent prior to participation in each of the surveys. Data were downloaded in one .xlsx-file (one row per participant, one column per variable) and data cleaning was implemented using the statistical software R as well as R Studio [1,2]. The wording of each item and its response options in English and German language can be found on the second sheet of the excel data file (name of the sheet: “codebook”; first sheet: “data”). Additionally, the wording of each item in English language is provided in the PDF file uploaded to the OSF available from: https://osf.io/4hsxe/.
Data format	Raw, filtered
Description of data collection	Inclusion criteria for participation in the online surveys: • Age ≥ 18 years • Eligibility to vote in the general German elections in 2021 • Giving informed electronic consent prior to participation For eligibility to participate in the surveys at T2 and T3, participants needed to participate in the initial survey (T1), first.
Data source location	Institution: Ulm University City: Ulm Country: Germany
Data accessibility	Repository name: Open Science Framework – osf.io Project identification number: DOI: 10.17605/OSF.IO/DR2PQ Direct URL to the dataset: https://osf.io/56z9k/

## Value of the Data

• The present data set is useful to further examine relations of, for example, sociodemographic variables, extraordinary events, and news consumption with voting intentions (cross-sectionally) as well as to investigate the stability (longitudinal data) of voting intentions, thus, political attitudes, in relation to the aforementioned variables.
• Political scientists as well as researchers from the field of psychology can profit from using and analyzing this data set.
• The present data set can provide first insights into associations of variables across time (e.g., frequency of being confronted with counter-attitudinal news when consuming political news, political attitudes, etc. across T1, T2, T3). These associations provide insights of importance to estimate the necessary sample sizes for forthcoming research projects.
• Additionally, content analyses on which kinds of extraordinary events impact voting intentions, thus, political attitudes, can be conducted.
• Finally, the present data on a German sample might contribute to cross-cultural comparisons of associations between various variables related to news consumption and political attitudes when combined with additional data sets from other countries.

## 1 Data Description

The relations between information consumption, more specifically political news consumption, and political attitudes gained increasing attention during the last decade. More specifically, some experts fear that a high degree of homogeneity (i.e., mostly attitude-aligning news) of political news consumption is contributing to extremization and polarization of political attitudes; see, for example, discussions on “filter bubble” and “echo chambers”. Oftentimes especially algorithms on the internet are being criticized for information filtering and contributing to extremization and polarization of opinions [3], [4], [5].

However, empirical research on the existence of absolutely homogeneous information environments is inconclusive [6], [7], [8], [9], [10], [11]; specifically since individuals can use various different online as well as offline news outlets. Similarly, also findings on the extent of associations between the degree of homogeneity versus heterogeneity of political news consumption and political attitudes [9,[12], [13], [14] are heterogeneous. Finally, the causal direction of relations between news consumption and political attitudes are unclear. Based on the heterogeneous findings as well as the lack of longitudinal data sets to investigate causal relations, the present data set was collected. It contains data of German individuals providing information on some control variables, such as sociodemographic variables, as well as on news consumption, and political attitudes longitudinally assessed at three assessment time points (T1, T2, T3).

After data cleaning (explained in detail below), the final data set comprises data of N = 122 individuals (n = 84 men, n = 38 women) who participated at least in the survey at T1 and the survey at T3. The mean age of the sample at T1 was M = 30.09 (SD = 12.53) years, and the mean age at T3 was M = 30.85 (SD = 12.61). Most participants indicated either high school degree/A-level (T1: n = 48, T3: n = 50) or a university, including university of applied sciences, degree (T1: n = 52, T3: n = 57) as their highest educational degree. Participants came from all federal states in Germany, except the Saarland and Saxony-Anhalt.

Items presented to all participants in the surveys at T1, T2, and T3 and for which data are included in the data set are presented in Table 1. References to the original publications of established measures, which were not specifically created by the project team for the present data assessments, are provided in this table as well.

**Table 1:** Items in the data set.

Survey	Items
T1	- Date of participation - Items on sociodemographic background (age, gender identity, highest educational degree, income, employment status, federal state) - Additional items on inclusion criteria (eligibility to vote in the general German elections in 2021, attention check item) - Item on which news channel is in general used most often to inform oneself of news about political issues - Item on which news channel is considered most trustworthy when it comes to complete, truthful and unbiased reporting of current news - Item on frequency of being presented with views that contradict own views when consuming news about political issues in general - Item on average time spent (in hours) to consume news per week in general - Item on stability of voting decisions for a specific party - Items on party affiliation [15] - “Sonntagsfrage” to assess voting intentions (based on Sindermann et al. [16]) - Left-right ideological self-placement [17] - Item on interest in politics [15]

T2	- Date of participation - “Sonntagsfrage” to assess voting intentions (based on Sindermann et al. [16]) - Item on which news outlet was most frequently used to inform oneself about political matters most recently (over the past month) - Item on frequency of having been presented with views that contradict own views when consuming news about political issues during the past month - Item on average time spent (in hours) to consume news per week during the past month - Items on whether any special event happened that changed one's voting intentions during the past month; if yes, what the event was

T3	- Date of participation - Items on sociodemographic background (age, gender identity, highest educational degree, income, employment status, federal state) - “Sonntagsfrage” to assess voting decisions in terms of first vote as well as second vote in the general German elections (based on Sindermann et al. [16]) - Items on most recent patterns of news consumption (during the past month): - Item on which news outlet was most frequently used to inform oneself of news about political issues during the past month until the general elections - Item on frequency of having been presented views that contradict own views when consuming news about political issues during the past month until the general elections - Item on average time spent (in hours) to consume news per week during the past month - Items on whether any special event happened that changed one's voting decision during the past month; if yes, what the event was - Retrospective items on the time between participation in the initial survey (T1) and the final survey (T3): - Questionnaire to assess the degree of homogeneity versus heterogeneity of political news consumption (HoHe) score [14] for the time between participation in the initial (T1) and the final survey (T3) - Item on which news outlet was most frequently used to inform oneself of news about political issues during the time between participation in the initial (T1) and the final survey (T3) - Item on which news outlet was considered most trustworthy when it comes to complete, truthful and unbiased reporting on current news for the time between participation in the initial (T1) and the final survey (T3) - Item on frequency of having been presented views that contradict own views when consuming news about political issues during the time between participation in the initial (T1) and the final survey (T3) - Item on average time spent (in hours) to consume news per week during the time between participation in the initial (T1) and the final survey (T3)

Note: The past month was used as time frame for “most recent” news consumption in order to get an impression of the general current/recent patterns of news consumption and to prevent non-generalizable/biased responses when only focusing on single days/weeks. Most items/measures were created by the research team for the present surveys; if items/measures from previous works were used, the respective reference is provided.

Data of all surveys and all items are included in the excel file (sheet: “data”) provided alongside this article. The excel file does also include detailed descriptions of the wording of each item and its response options in both German and English language (sheet: “codebook”). Additionally, the wording of each item in English language is provided in the PDF file uploaded to the OSF available from: https://osf.io/4hsxe/.

**Fig. 1:**
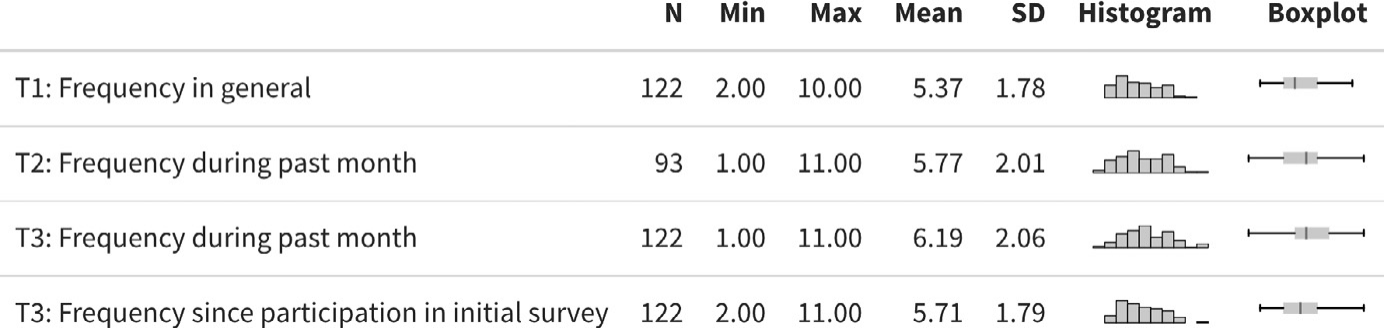
Descriptive statistics on frequency of being presented with views that contradict own views when consuming news about political issues assessed at T1, T2, and T3 (possible range of responses: 1-11).

**Fig. 2:**
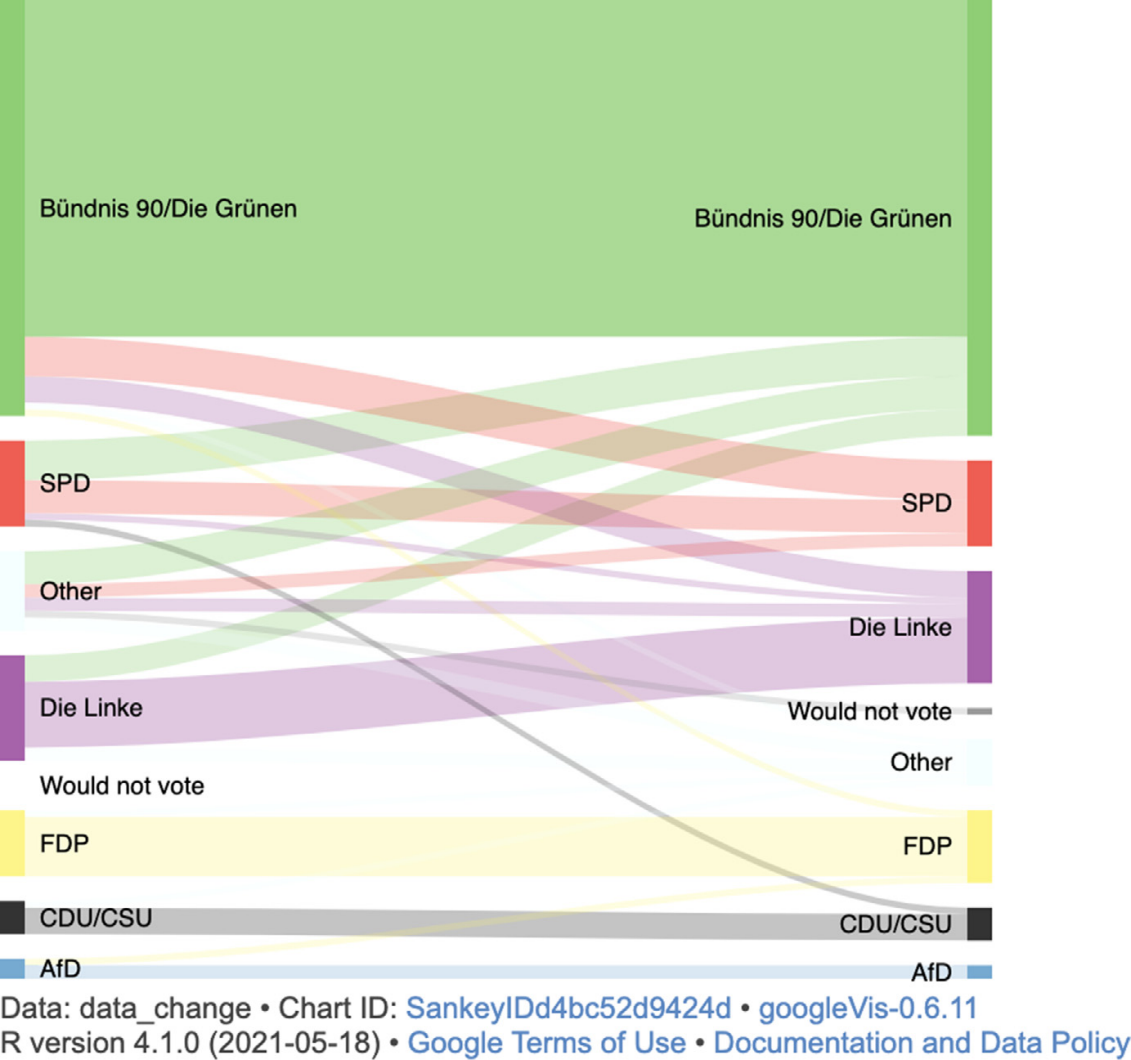
Sankey diagram on voting intentions/decisions from T1 to T3 (second vote) on individual level. The graph was built in the statistical software R [1] and R Studio [2] using the googleVis package [19]. The code of an interactive html version is available from: https://osf.io/r6yfv/.

Fig. 1 and Table 2 contain information on the distributions of the variables on being confronted with views contradicting one's own views when consuming political news (Fig. 1) and on voting intentions/decisions (Table 2) across T1, T2, and T3. Fig. 2 additionally provides an illustration of voting stability/change of individuals from T1 to T3. The html code for the interactive plot related to Fig. 2 is available from: https://osf.io/r6yfv/.

**Table 2:** Descriptive statistics of voting intentions at T1 and T2, and actual voting decisions provided at T3.

	T1		T2		T3			
	Voting intentions		Voting intentions		Voting decision: First vote		Voting decision: Second vote	
	N	%	N	%	N	%	N	%
Die Linke	16	13.11	7	7.53	15	12.30	17	13.93
SPD	13	10.66	5	5.38	26	21.31	13	10.66
Bündnis 90/Die Grünen	63	51.64	57	61.29	56	45.90	66	54.10
FDP	10	8.20	11	11.83	8	6.56	11	9.02
CDU/CSU	5	4.10	6	6.45	9	7.38	5	4.10
AfD	3	2.46	2	2.15	2	1.64	2	1.64
Other	12	9.84	2	2.15	5	4.10	7	5.74
Would not vote	0	0.00	3	3.23	1	0.82	1	0.82

Note: German parties are ordered from left (Die Linke = The left) to right (AfD = Alternative for Germany) according to Volkens et al. [18]; SPD = Social Democratic Party of Germany, Bündnis 90/Die Grünen = Alliance 90/Greens, FDP = Free Democratic Party, CDU/CSU = Christian Democratic Union/Christian Social Union. If percentages do not add up to exactly 100%, this is due to rounding.

## 2 Experimental Design, Materials, and Methods

**Fig. 3:**
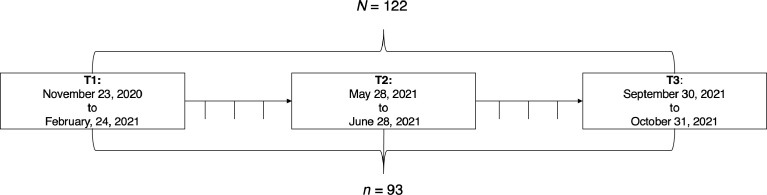
Timeline of data collection (official start and end date of data collection per survey) and final sample sizes in the data set.

The present data set was collected from a sample of German volunteers eligible to vote in the general German elections in 2021. More specifically, all participants included in the data set participated in an initial survey (T1) between November 2020 and February 2021. After participating in this survey, participants were re-invited to participate in monthly surveys starting in the end of February until after the general German elections in September 2021. Invitations were sent to all participants who provided their email address after participating in the initial survey (T1) on a monthly basis via email. Unfortunately, due to issues related to data matching and issues when programming the survey, only data from two additional surveys after the initial survey (T1) can be provided: Data from the survey (T2) conducted from end of May to end of June 2021 and data from the final survey (T3) after the general elections in Germany in 2021, which was conducted between end of September 2021 and October 2021. A summary of the data collection is depicted in Fig. 3. One of the aforementioned issues and, hence, one reason for restriction in the provision of data (i.e., providing data of only three surveys) is related to issues with data matching across the monthly surveys. More specifically, to match data across surveys, participants needed to provide an individual code in the beginning of participation in every survey. Unfortunately, there were many data sets of individuals who apparently provided different individual codes in different surveys and whose data could not be matched, accordingly. Additionally, another reason why only data of three surveys is provided is related to a programming error (by the researchers, not related to the functionality of the survey platform) which led to the item on voting intentions only being shown to few participants in the first three monthly surveys.

Participation in the initial survey was advertised in various ways both offline (printed press, etc.) and online (social media, online magazines, etc.): When the first author gave an interview on topics related to the content of the survey, the link was presented. Additionally, Twitter ads were used, and students writing their theses on data of this research project supported data collection. Lastly, some influencers on Instagram and YouTube provided the link to the survey to their audiences and followers.

All surveys were programmed on the SurveyCoder tool (https://www.surveycoder.com/; https://ckannen.com/). As an incentive for participation, all participants received anonymous feedback on their scores in several of the self-report measures assessed. The local ethics committee at Ulm University, Ulm, Germany approved the data collection including all monthly surveys. The data set including the exact wording of all items and all response options in English and German language is provided in the excel data file. Aside from sociodemographic variables, which were used in previous studies, items on party affiliation [15], the “Sonntagsfrage” to assess voting intentions (based on Sindermann et al. [16]), the item on left-right ideological self-placement [17], and the item on interest in politics [15], all items used in the survey at T1 were created by the research team specifically for this survey. At T2, the “Sonntagsfrage” (based on Sindermann et al. [16]) was the only measure, which was not created by the research team. At T3, sociodemographic variables, the “Sonntagsfrage” (based on Sindermann et al. [16]) and the questionnaire to assess the degree of homogeneity versus heterogeneity of political news consumption (HoHe) score [14] were the only measures derived from previous works. Researchers interested in building the HoHe score from the present data can inspect the detailed descriptions and the formula presented in Sindermann et al. [14] in order to receive information on how to compute this composite score. Other than this score, measures included in the surveys are single item measures and/or have not been collapsed to composite scores before.

The present data set contains data of individuals who completed the surveys at T1 and T3. Data from the survey at T2 are also provided for those participants who participated in the survey at T2 and whose data could be matched to their T1 and T3 survey data. Participants not participating in both the T1 and T3 survey are not included in the data set.

During data cleaning, none of the participants completing both the T1 and T3 survey needed to be excluded due to missing data. Data of n = 1 participant were excluded due to the participant stating to be younger than 18 years. Additionally, data of n = 2 participants were excluded because their gender provided in the initial survey did not match the gender provided in the final survey; thus, two people of different gender might have provided the same code and their data sets have wrongfully been matched, accordingly. Next, n = 2 participants were excluded due to implausible age specifications across the initial and the final survey (i.e., providing a younger age in the final survey compared to the initial survey or aging more than one year), and n = 2 additional participants were excluded due to providing a higher educational degree in the initial compared to the final survey. Finally, n = 9 participants were excluded due to failing the attention check item. This led to the final sample size of N = 122 participants.

## Ethics Statements

The present research project was approved by the local ethics committee of Ulm University, Ulm, Germany (247/20). The data collection procedure followed the latest revision of the Declaration of Helsinki. All participants provided informed electronic consent prior to participation in each of the surveys. Data were collected in a pseudonymous way to match data across surveys. Data were anonymized before the upload.

## CRediT Author Statement

**Cornelia Sindermann:** Conceptualization, Methodology, Software, Validation, Formal Analysis, Investigation, Data Curation, Writing – original draft, Visualization, Supervision, Project Administration, Funding Acquisition; **Christopher Kannen:** Software, Writing – review & editing; **Christian Montag:** Writing – review & editing.

## Declaration of Competing Interest

The authors declare that they have no known competing financial interests or personal relationships that could have appeared to influence the work reported in this paper.

Nevertheless, the authors declare the following financial interests/personal relationships:

For reasons of transparency Dr. Montag mentions that he has received (to Ulm University and earlier University of Bonn) grants from agencies such as the German Research Foundation (DFG). Dr. Montag has performed grant reviews for several agencies; has edited journal sections and articles; has given academic lectures in clinical or scientific venues or companies; and has generated books or book chapters for publishers of mental health texts. For some of these activities he received royalties, but never from gaming or social media companies. Dr. Montag mentions that he is part of a discussion circle (Digitalität und Verantwortung: https://about.fb.com/de/news/h/gespraechskreis-digitalitaet-und-verantwortung/) debating ethical questions linked to social media, digitalization and society/democracy at Facebook. In this context, he receives no salary for his activities. Finally, he mentions that he currently functions as independent scientist on the scientific advisory board of the Nymphenburg group (Munich, Germany). This activity is financially compensated. Moreover, he is on the scientific advisory board of Applied Cognition (Redwood, CA, USA), an activity which is also compensated.

## Data Availability

data_Sindermann_et_al_longit_OSF_clean_2022_03_16.xlsx (Original data) (Open Science Framework).

## Appendix Supplementary materials

Image, application 1
